# Fracture Resistance of Endodontically Treated Teeth Prepared With Biologically Oriented Preparation Technique Versus Horizontal Finish Lines: An In Vitro Study

**DOI:** 10.7759/cureus.73447

**Published:** 2024-11-11

**Authors:** S K Jagdish, Rajamony Eazhil, Karnan Vijila Kaviya, Kaleel Imra, Govinda Raj Sarathchandra

**Affiliations:** 1 Department of Prosthodontics and Implantology, Chettinad Dental College and Research Institute, Kelambakkam, IND; 2 Department of Prosthodontics, Rajas Dental College and Hospital, Kavalkinaru, IND

**Keywords:** biologically oriented preparation technique, endodontically treated teeth, fracture resistance, horizontal preparation, monolithic zirconia crowns

## Abstract

Introduction: The biologically oriented preparation technique (BOPT) is a conservative tooth preparation method with no defined finish lines. Studies comparing the fracture resistance of endodontically treated teeth (ETT) prepared using BOPT or horizontal finish lines are lacking. The primary objective of this study was to compare the fracture resistance of ETT prepared using BOPT, chamfer finish line, or shoulder finish line and restored with monolithic zirconia (MZ) crowns. The secondary objective was to determine the modes of fracture of ETT with different finish lines.

Methods: Forty-five maxillary premolar teeth were endodontically treated and randomly allotted to three tooth preparation methods: Group 1, BOPT; Group 2, chamfer finish line; and Group 3, shoulder finish line. MZ crowns were milled and cemented on their respective tooth preparations. The fracture resistance was tested using a universal testing machine. Maximum fracture load was recorded in Newtons (N). The fracture modes were classified using Burkey's codes and a newly proposed fracture grading.

Results: The highest fracture resistance was seen in samples from Group 1, followed by Group 2, and the least in Group 3. Tukey's post hoc test showed a significant difference between Group 1 and Group 3 (p<0.05) and between Group 2 and Group 3 (p<0.05). No significant differences were found between Group 1 and Group 2 (p>0.05). Code V and Grade 3B fractures were highest in Group 3 samples.

Conclusions: Within the limitations of the present study, it can be concluded that ETT prepared with BOPT and chamfer finish lines had the greatest fracture resistance compared to shoulder finish lines. Teeth prepared with shoulder finish lines had more non-restorable fractures compared to BOPT or chamfer preparations.

## Introduction

Endodontically treated teeth (ETT) have compromised tooth structure and are more prone to fracture than vital teeth. The prognosis of ETT depends on the remaining coronal tooth structure after the endodontic therapy [1]. Post-endodontic restoration aims to preserve and reinforce the remaining tooth structure and prevent non-restorable fractures. Numerous direct and indirect post-endodontic restorations have been used to restore ETT [2]. Direct restorative options include dental amalgam, conventional composites, fiber-reinforced composites, and post and core systems. With advancements in adhesive dentistry, indirect treatment options such as inlays, onlays, endocrowns, partial crowns, or full crowns are also used with predictable clinical outcomes. The short-term (2.5-3 years) prognosis of direct and indirect restorative options for ETT is comparable, with no significant difference in their clinical outcomes [3]. However, the 5- to 10-year prognosis is higher for indirect restorations, particularly full crowns [4]. A recent umbrella review suggested that full crowns are more likely to be the proper treatment option for ETT when compared to other prosthetic restorations [5].

Monolithic zirconia (MZ) crowns are becoming increasingly popular as full-coverage restorations because of their favorable material properties. MZ crowns require minimal tooth preparation, which preserves residual tooth structure in the ETT [6]. With no veneering material, MZ crowns display fewer mechanical failures such as chipping or cracks [7]. Computer-aided designing and milling (CAD/CAM) has significantly improved the marginal fit and adaptation of MZ crowns [8]. Clinical trials using MZ crowns have shown favorable gingival and periodontal responses [9]. All these factors make the MZ crown an ideal post-endodontic restoration for ETT.

Tooth preparation for prosthetic crowns may be done using horizontal or vertical finish lines. Horizontal finish lines such as the shoulder, chamfer, or deep chamfer are preparations with a defined margin [10]. In contrast, vertical tooth preparations do not have well-defined margins and are usually indicated for periodontally compromised teeth [11]. Few authors refer to vertical tooth preparations with terms such as shoulderless, knife edge, or slice preparations [12-15]. Loi and Di Felice described a modified vertical preparation protocol called the biologically oriented preparation technique (BOPT) that can be used with zirconia or ceramometal restorations [11]. BOPT is a highly conservative tooth preparation method that is simple and easy to prepare compared to other finish line designs. In contrast to other vertical finish lines, the BOPT typically eliminates the cementoenamel junction (CEJ) and extends into the sulcus by 0.5-1 mm. BOPT also involves controlled removal of the gingival sulcus epithelium (gingitage) followed by immediate temporization. This technique permits the positioning of restoration margins at different levels without affecting marginal adaptation, emergence profile, and quality of the soft tissues [11]. The effect of finish line configurations on the fracture resistance of the teeth remains unclear. Many studies have claimed that the type of finish line influences the fracture resistance of MZ crowns [12-20], while few reports claim otherwise [21-23].

Literature on the effect of BOPT or horizontal finish lines on the fracture resistance of ETT restored with MZ crowns is scarce. The primary objective of this study was to compare the fracture resistance of ETT prepared using BOPT, chamfer finish line, or shoulder finish line and restored with MZ crowns. The null hypothesis was that the type of finish line did not influence the fracture resistance of ETT restored using MZ crowns. The secondary objective was to determine the modes of fracture of ETT with different finish lines.

## Materials and methods

An in vitro study was designed to analyze the fracture resistance of endodontically treated maxillary premolars prepared using three finish line designs and restored with MZ crowns. The study protocol was approved by the Institutional Human Ethics Committee (IHEC-CDCRI/2024/FAC-0032). Informed and written consent was waived as only non-identifiable biological wastes (extracted teeth) were collected.

Sources of samples and sample size estimation

Maxillary premolar teeth extracted from patients undergoing orthodontic treatment aged between 18 and 25 years were used in this study. Sample size estimation was done using statistical software (G*Power, version 3.1.9.4, Heinrich-Heine-Universität Düsseldorf, Düsseldorf, Germany). The effect size (f=0.54) was determined using the mean and standard deviation (SD) of fracture resistance of MZ crowns obtained from a previous study [16]. A sample size of 39 was estimated using an alpha error of 0.05 (95% confidence) and 80% power. The final sample size for the three groups was increased to 45 (15 samples per group). The extracted teeth were cleaned with an ultrasonic scaler (UDS-J Ultrasonic Scaler, Guilin Woodpecker Medical Instrument Co. Ltd., Guangxi, China) and stored in 0.9% saline to prevent dehydration. All the teeth were screened using the Decayed, Missing, and Filled Teeth (DMFT) Index for caries and restorations. Tooth fractures, chipping, and discoloration were examined using a stereomicroscope (Luxeo 4X Microscope, Labomed Inc., Los Angeles, CA). Teeth with fracture, caries, discoloration, restorations, insufficient crown-to-root ratio, crown or root malformation, and short clinical crowns were excluded from the study.

Root canal instrumentation and obturation

An endodontic access cavity was prepared using a round bur (BR-31C, Mani, Inc., Utsunomiya, Japan). The working length was measured by introducing a No.10 K-file (Mani, Inc., Utsunomiya, Japan) in the canals until it was seen from the apical foramen. The final working length was obtained by subtracting 1 mm from this length. Root canal instrumentation was done using rotary Ni-Ti files (Protaper Gold, Dentsply, Zurich, Switzerland) with an endo motor (X-Smart, Dentsply, Zurich, Switzerland) in a crown-down manner using gentle in-and-out motion. The canals were shaped using the S1 and S2 files and finished with F1 and F2 files to the full working length. Between each file, irrigation was done using 2 mL of 5.25% sodium hypochlorite followed by 2 mL of 0.9% saline per root canal. A total of 20 mL of irrigant was used per canal during the mechanical preparation. The canals were dried and obturated using the single cone technique with matched gutta-percha points (Dentsply, Zurich, Switzerland) and hydraulic sealer (Bio C-Sealer, Angelus, Brazil).

Restoration of access cavity

The excess gutta-percha was removed from the pulp chamber till the root canal orifices. The access cavity was selectively etched using 37% phosphoric acid gel (Total Etch, Ivoclar, Schaan, Liechtenstein) for 10 seconds and washed with water. A bonding agent (Tetric N-Bond, Ivoclar, Schaan, Liechtenstein) was applied, and the access cavity was restored using a composite resin (Multicore Flow, Ivoclar, Schaan, Liechtenstein) and light polymerized for 20 seconds. The teeth were then mounted in cold cure acrylic resin (DPI RR Cold Cure, Dental Products of India, Uttarakhand, India) using preformed rubber molds with the help of a dental surveyor (Unident Dental Surveyor, New Delhi, India). All the teeth were oriented parallel to the long axis and embedded in the acrylic blocks 1 mm apical to the cementoenamel junction (CEJ).

Randomization and tooth preparation

The teeth were then randomly allotted to three groups using a lottery method as follows: Group 1, teeth prepared with the BOPT; Group 2, teeth prepared with a 0.5 mm chamfer finish line; and Group 3, teeth prepared with a 1 mm shoulder finish line. The allocation ratio was 1:1:1 resulting in 15 samples per group. The dimensions of the crowns of the teeth (mesiodistal width, buccolingual width, and height) allotted to the three groups were measured at baseline using a digital vernier caliper (Skadioo Digital Vernier Caliper, Perfect Sales India, Haryana, India).

**Figure 1:**
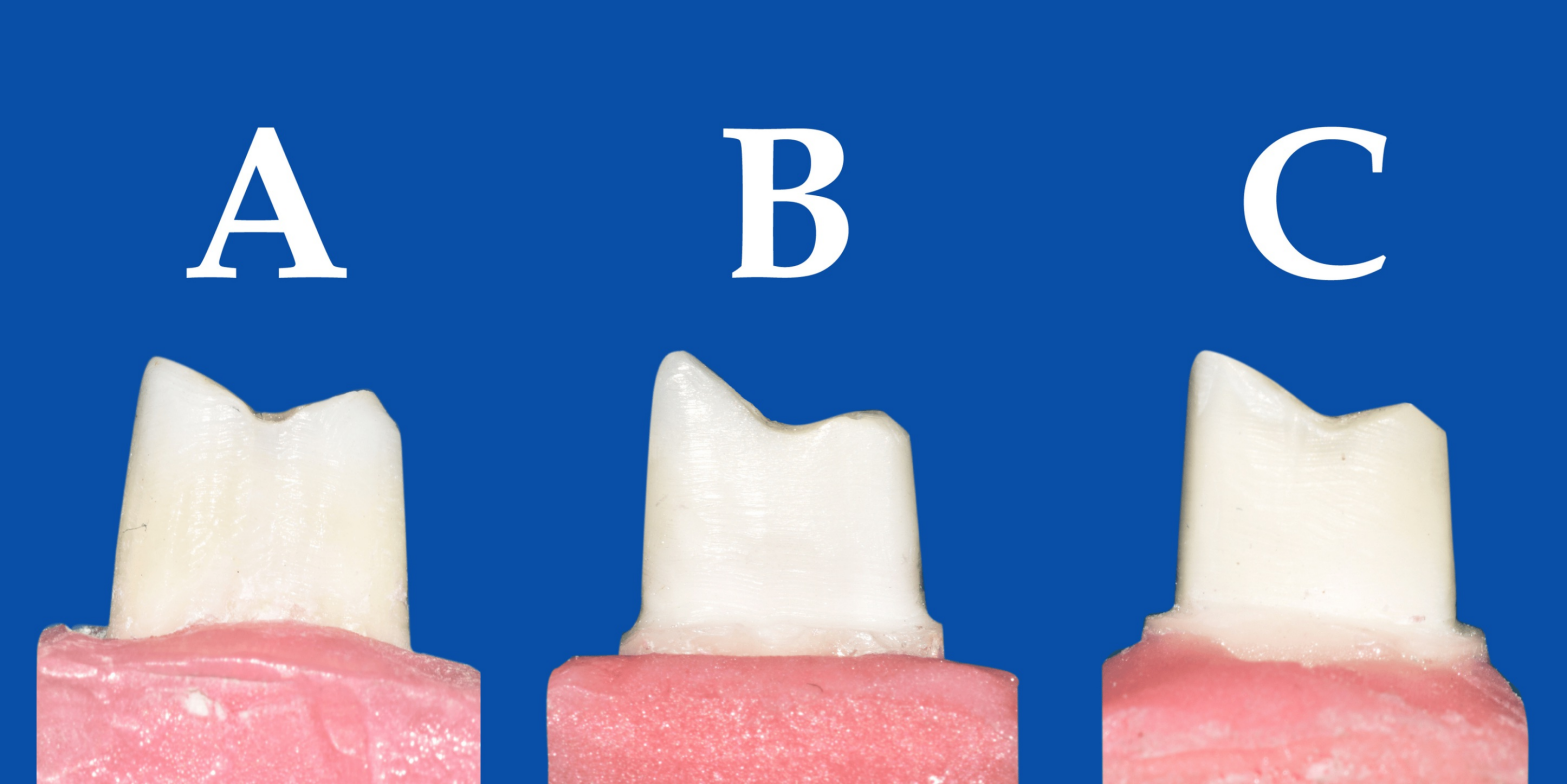
Tooth preparation in three groups A: Group 1 (BOPT), B: Group 2 (chamfer finish line), C: Group 3 (shoulder finish line) BOPT: biologically oriented preparation technique

Tooth preparations were done by a single operator using a high-speed airotor handpiece and water coolant. Before preparation, a putty index of each tooth was made. The depth of preparation was measured using a William marking probe (POW6#6, GDC Fine Crafted Dental Pvt. Ltd., Hoshiarpur, India). In all the groups, a 1 mm occlusal reduction was done using a barrel-shaped diamond bur (EX-12, Mani, Inc., Utsunomiya, Japan). Tooth preparation in Group 1 was done according to the BOPT described by Loi and Di Felice [11]. The axial preparation was done using a coarse (green) point-end tapered diamond bur (TC-11C, Mani, Inc., Utsunomiya, Japan) and extended to 1 mm below the CEJ with a total 6° occlusal convergence. The preparations were finished using a fine (red) point-end tapered diamond bur (TC-11F, Mani, Inc., Utsunomiya, Japan). The samples in Group 2 were prepared with a 0.5 mm chamfer finish line using a coarse (green) round-end tapered diamond bur (TR-13C, Mani, Inc., Utsunomiya, Japan) with an occlusal convergence of 6°. Finishing was done using a fine (red) round-end tapered diamond bur (TR-13F, Mani, Inc., Utsunomiya, Japan). The samples in Group 3 were prepared with a 1 mm shoulder finish line using a coarse (green) flat-end tapered diamond bur (TF-13C, Mani, Inc., Utsunomiya, Japan) with an occlusal convergence of 6°. Finishing was done using fine (red) flat-end tapered diamond bur (TF-12F, Mani, Inc., Utsunomiya, Japan). The sample tooth preparations in the three groups are shown in Figure 1.

Fabrication of crowns and cementation

The prepared teeth were digitally scanned using a laboratory scanner (T 510 3D Scanner, Medit Corp., Seoul, South Korea). MZ crowns were designed using CAD software (DentalCAD 3.0 Galway, Exocad GmbH, Darmstadt, Germany) with an 80 µm spacer and 1 mm occlusal thickness. The crowns were milled on zirconia blocks (ceraMotion Z HT Shade, Dentaurum GmbH & Co. KG, Ispringen, Germany) using a 5-axis milling machine (5X-300 Pro, Aurum Dentistry, Daejeon, South Korea). The crowns were sintered (Tabeo 1/M/ZIRKON-100 Sintering furnace, Mihm-Vogt GmbH & Co KG, Blankenloch, Germany) at 1530°C, followed by a cooling cycle according to the build-in program in the sintering furnace.

The internal surfaces of the crowns were sandblasted using 50 µm alumina particles, cleaned with water, and air-dried. The crowns were evaluated for marginal adaptation, form, and contour according to the revised FDI World Dental Federation (FDI) criteria [24]. Only clinically acceptable crowns (excellent or good) were used in this study. Crowns were cemented on their respective teeth using a self-adhesive resin cement (Rely X U200, 3M ESPE, Seefeld, Germany). The crowns were first cemented using finger pressure, followed by applying a 5 kg vertical load for six minutes using a custom-made device. A piece of rubber was placed on the occlusal surface of the crowns during cementation to avoid direct contact and simulate a cotton roll effect [18]. All the samples were subjected to a thermocycling process in a water bath between 5°C and 55°C for 500 cycles at an interval of 30 seconds.

Fracture testing

The fracture resistance of all the samples was tested using a universal testing machine (UTM M-100, Fine Spavy Associates & Engineers, Maharashtra, India). The samples were mounted on the universal testing machine using a customized clamp. Vertical load was applied at the central fossa of the crowns using a 13 mm round-end stainless steel indenter with a cross-head speed of 0.5 mm per minute. A 2 mm thick rubber material was placed between the indenter and the crowns to prevent direct contact [18]. The load was applied till the point of fracture of the crown or the tooth. The maximum fracture load was recorded in Newtons (N). Fractured samples were then collected and analyzed under magnification using a stereomicroscope (Luxeo 4X Microscope, Labomed Inc., Los Angeles, CA).

Classification of fracture modes

**Figure 2:**
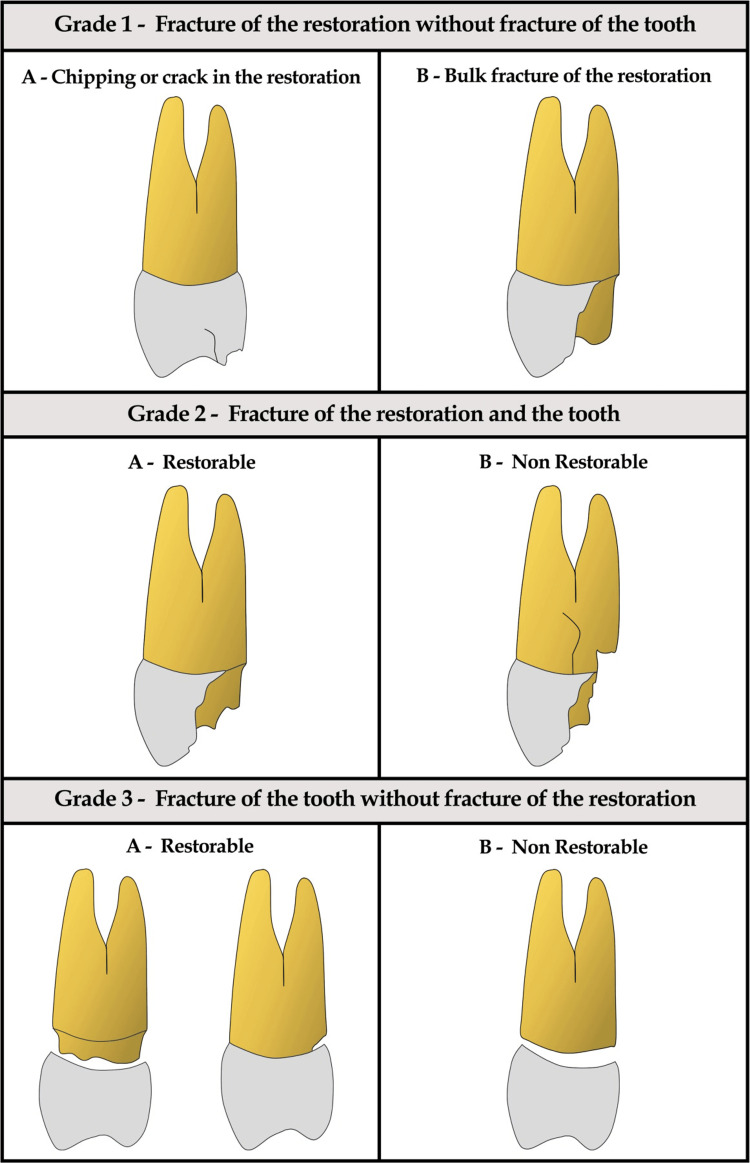
Newly proposed fracture grading for crown/tooth fractures

The types of fractures were classified according to Burke's codes [25] as shown in Table 1. Alternatively, a newly proposed fracture grading was also used to classify the fracture modes. This new grading system classified the fractures into three grades: Grade 1, fractures of the restoration without fracture of the tooth; Grade 2, fracture of the restoration and the tooth; and Grade 3, fracture of the tooth without fracture of the restoration. Each grade was further classified into sub-classes A and B. Fractures above the CEJ with a minimum of 2-3 mm of tooth structure were classified as restorable. Those fractures that extended below the CEJ and into the root were classified as non-restorable. Representative diagrams with descriptions for the three grades and sub-classes are shown in Figure 2.

**Table 1:** Codes used in assessing the extent of fracture Source: Burke FJ: Maximising the fracture resistance of dentine-bonded all-ceramic crowns. J Dent. 1999, 27:169-73. 10.1016/s0300-5712(98)00050-5 [25]

Code	Description
Code I	Minimal fracture or crack in the crown
Code II	Less than half of the crown lost
Code III	Crown fracture in the midline; half of the crown displaced or lost
Code IV	More than half of the crown lost
Code V	Severe fracture of the tooth and/or crown

Statistical analysis

Descriptive and inferential statistics were analyzed using IBM SPSS version 20.0 (IBM Corp., Armonk, NY). Mean and standard deviation (SD) were used to summarize the dimensions and fracture loads. The Shapiro-Wilk test was used to test the normality of the dataset. An intragroup comparison was done using a one-way analysis of variance (ANOVA), and when significant, a Tukey's post hoc test was used for intragroup pairwise comparisons. Frequency and percentage were used to summarize the category of fractures under Burke's classification and the new fracture grading. A p value of <0.05 was considered a statistically significant difference.

## Results

The Shapiro-Wilk test showed that all the data were normally distributed; hence, parametric tests were used for intergroup comparisons. There were no significant (p>0.05) differences in the baseline dimensions of the teeth between the three groups (Table 2).

**Table 2:** Comparison of crown dimensions of teeth between the three groups at baseline One-way ANOVA was used for the intergroup comparisons. Group 1: teeth prepared with BOPT, Group 2: teeth prepared with chamfer finish line, Group 3: teeth prepared with shoulder finish line SD: standard deviation, ANOVA: analysis of variance, BOPT: biologically oriented preparation technique

Parameter	Group	Mean	SD	F	p value
Mesiodistal width (in mm)	Group 1	7.15	0.42	0.029	0.971
	Group 2	7.14	0.39		
	Group 3	7.12	0.33		
Buccolingual width (in mm)	Group 1	9.03	0.69	0.105	0.901
	Group 2	8.98	0.68		
	Group 3	9.09	0.67		
Height (in mm)	Group 1	7.03	0.36	0.335	0.717
	Group 2	7.09	0.26		
	Group 3	7.11	0.19		

The highest fracture resistance was seen in samples from Group 1 (2457.67±101.21 N), followed by Group 2 (2365.20±119.36 N), and least in Group 3 (1865.47±81.26 N). Comparison of fracture resistance of samples using one-way ANOVA showed a statistically significant difference (p<0.05) between the three groups (Table 3). Tukey's post hoc test showed a significant difference between Group 1 and Group 3 (p<0.05) and between Group 2 and Group 3 (p<0.05). No significant differences were found between Group 1 and Group 2 (p>0.05).

**Table 3:** Comparison of fracture resistance of samples between the three groups One-way ANOVA was used for the intergroup comparisons. Group 1: teeth prepared with BOPT, Group 2: teeth prepared with chamfer finish line, Group 3: teeth prepared with shoulder finish line * indicates values with statistically significant difference (p<0.05). Same lowercase letter denotes a statistically significant difference in Tukey's post hoc test for multiple pairwise comparisons. N: Newton, SD: standard deviation, ANOVA: analysis of variance, BOPT: biologically oriented preparation technique

Parameter		Mean	SD	F	p value
Fracture load (in N)	Group 1	2457.67^a^	101.21	146.9	0.0001*
	Group 2	2365.20^b^	119.36		
	Group 3	1865.47^ab^	81.26		

Classification of fractures according to Burkey's codes showed that fracture of the crown and/or the tooth (Code V) was the most common mode of failure in all three groups. Code III, Code IV, or Code V fractures were seen in Group 1 and Group 2 samples. All the samples in Group 3 had only Code V fractures. Code V fractures were highest in Group 3 (100%), followed by Group 2 (80%), and least in Group 1 (67.7%). None of the samples had Code I or Code II fractures (Table 4).

**Table 4:** Descriptive statistics for modes of fracture using Burke's codes (1999) Group 1: teeth prepared with BOPT, Group 2: teeth prepared with chamfer finish line, Group 3: teeth prepared with shoulder finish line Values are expressed as percentages. BOPT: biologically oriented preparation technique, n: number of samples

Burkey's code	Group 1	Group 2	Group 3
	% (n)	% (n)	% (n)
I	-	-	-
II	-	-	-
III	13.3 (2)	6.7 (1)	-
IV	20 (3)	13.3 (2)	-
V	66.7 (10)	80 (12)	100 (15)
Total	100 (15)	100 (15)	100 (15)

**Figure 3:**
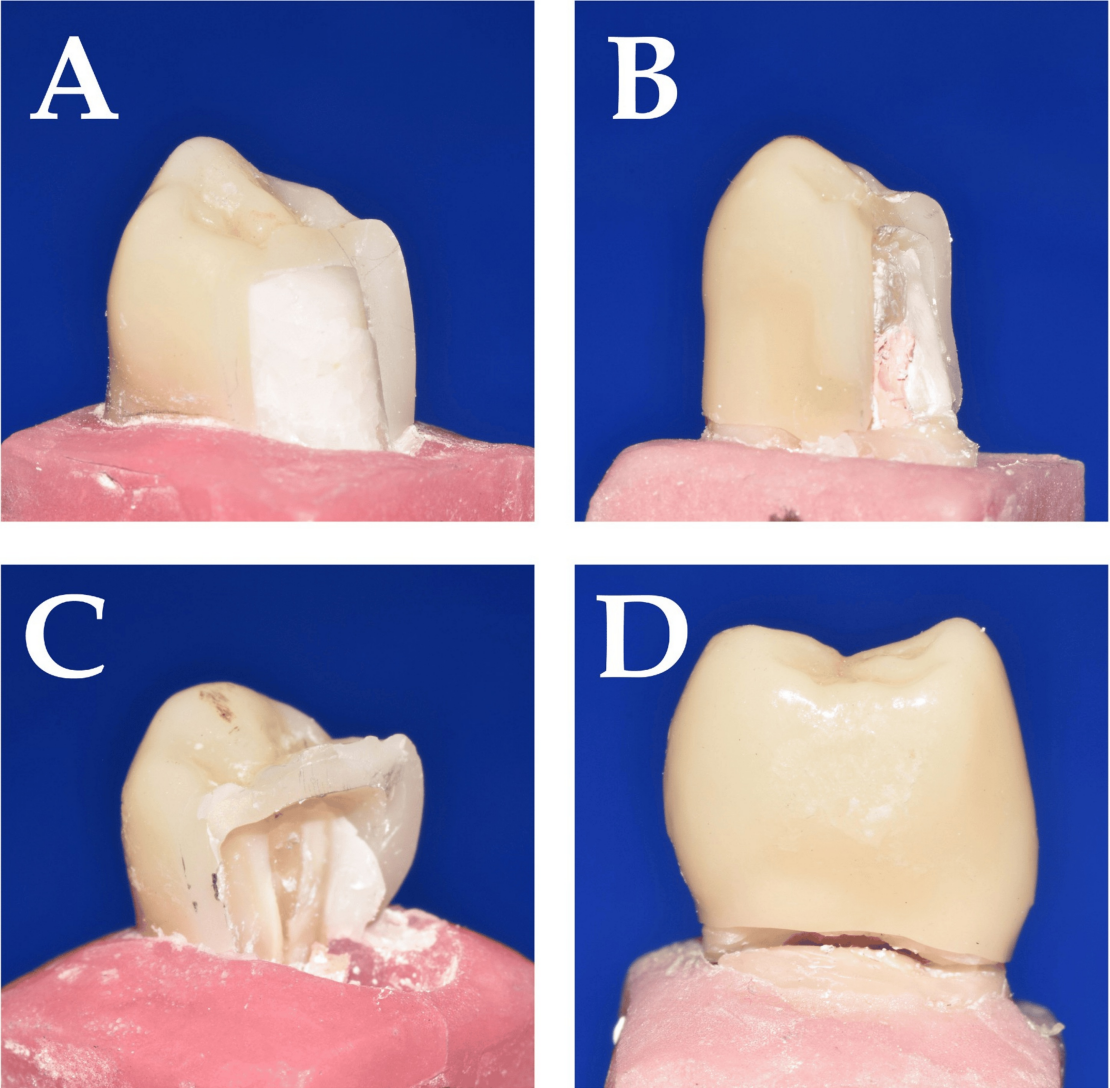
Sample images of observed fractures A: Burkey's Code III or Grade 1B fracture, B: Burkey's Code V or Grade 2A fracture, C: Burkey's Code V or Grade 2B fracture, D: Burkey's Code V or Grade 3B fracture

According to the new fracture grading, the maximum non-restorable fractures (73.3%) were seen in Group 3 (Grade 2B: 33.3%, Grade 3B: 40%). In contrast, 80% of Group 1 samples (Grade 1B: 33.3%, Grade 2A: 46.7%) and 73.3 % of Group 2 samples (Grade 1B: 20%, Grade 2A: 53.3%) had restorable fractures. Grade 3B fractures were seen only in Group 3. None of the samples in all three groups had Grade 1A or Grade 3A fractures (Table 5). Sample images of the modes of fractures observed in our study are shown in Figure 3.

**Table 5:** Descriptive statistics for modes of fracture using the newly proposed grading Group 1: teeth prepared with BOPT, Group 2: teeth prepared with chamfer finish line, Group 3: teeth prepared with shoulder finish line Values are expressed as percentages. BOPT: biologically oriented preparation technique, n: number of samples

Grade of fracture	Group 1	Group 2	Group 3
	% (n)	% (n)	% (n)
1A	-	-	-
1B	33.3 (5)	20 (3)	-
2A	46.7 (7)	53.3 (8)	26.6 (4)
2B	20 (3)	26.7 (4)	33.3 (5)
3A	-	-	-
3B	-	-	40 (6)
Total	100 (15)	100 (15)	100 (15)

## Discussion

In this in vitro study, we compared the fracture resistance of endodontically treated maxillary premolars prepared using three finish line designs and restored with MZ crowns. Our findings indicate that teeth with BOPT and chamfer finish lines had higher fracture resistance than teeth with shoulder finish lines. Thus, our null hypothesis was rejected. The teeth with shoulder finish lines had more non-restorable fractures, while those with BOPT or chamfer finish lines had more restorable fractures.

The outcomes of in vitro fracture tests of crowns are influenced by various factors such as choice of substrate material, experimental conditions, artificial aging, bonding protocols, and the type of crown material [16,18]. Among all these factors, the elastic modulus of the supporting substrate has been found to significantly influence the fracture resistance of all ceramic crowns [26]. Supporting materials such as natural teeth or substrates with low elastic modulus are more suitable for fracture tests as they simulate clinical conditions [26]. Using natural teeth also permits the bonding of all ceramic crowns to dentin [18]. Due to these advantages, we preferred using natural teeth as substrate instead of metal or resin dies.

Despite our best efforts, we could not find studies on fracture resistance of ETT prepared with BOPT or horizontal finish lines and restored with MZ crowns. Therefore, we propose that this will be the first in vitro study to evaluate the effect of finish lines on the fracture resistance of ETT. Our study is clinically relevant as single crowns are more commonly used to restore ETT than intact teeth in clinical practice. Although there were no similar studies, we have tried to compare the outcomes of a few reports that have used MZ crowns cemented to non-endodontically treated teeth (non-ETT) or other substrate materials.

Numerous studies have used metal or resin dies as substrates to test fracture loads of zirconia crowns or copings with different finish lines [12-15,17,19,20,23]. Despite the differences in the substrate materials, the findings of this study are concurrent with Jasim et al. [12] and Mitov et al. [13] who demonstrated that zirconia crowns with shoulderless preparations had higher fracture resistance than chamfer preparations. Our study is also consistent with Gavara et al. who found no difference in the fracture resistance between BOPT and chamfer finish line [20]. They reported higher fracture resistance with BOPT and deep chamfer finish lines than in shoulder preparations.

Our results are inconsistent with the findings of a few studies that have used metal or resin substrates [14,15,17]. Beuer et al. reported higher fracture resistance with shoulder preparations than shoulderless or chamfer preparations [14]. Similar findings by Jalalian et al. showed that fracture resistance was higher with a 1 mm deep chamfer compared to 0.8 mm chamfer preparation [17]. These studies have used zirconia copings instead of MZ crowns, which could have led to the differences observed. Skjold et al. reported that crowns with chamfer preparations fractured at higher loads than those with slice (shoulderless) preparations [15]. However, they used bilayer zirconia crowns with ceramic veneers instead of MZ crowns.

Only a few studies have used natural teeth as substrates to analyze the effect of finish lines on fracture resistance [16,18,21,22]. Aboushelib compared the fracture resistance of zirconia copings with complete ledge or chamfer finish lines and found no difference [21]. Kasem et al. reported higher fracture resistance in MZ crowns with vertical preparations (1347.6±177.4 N) than horizontal preparations (1255.6±121.3 N) [22]. However, the difference was insignificant. The fracture load values reported by Kasem et al. are much lower than those observed in this study [22]. This might be due to differences between the material properties of the zirconia blocks.

Findakly and Jasim [18] and Abdulazeez and Majeed [16] used MZ crowns with different finish line designs bonded to natural tooth substrate using the same adhesive cement used in this study (Rely X U200, 3M ESPE, Seefeld, Germany). Findakly and Jasim reported higher fracture loads in MZ crowns with 1 mm shoulder finish lines compared to feather edge finish lines [18]. Abdulazeez and Majeed found that MZ crowns with 0.8 mm chamfer had higher fracture resistance than those with vertical preparations [16]. Despite similar bonding protocols, substrates, and crown material, the results from both these studies contradicted our findings. The difference observed could probably be due to the use of non-ETT teeth as substrates in these studies, as opposed to the ETT. The shoulder preparations on ETT could have led to more loss of tooth structure compared to BOPT or chamfer finish lines resulting in lower fracture resistance.

Regarding the modes of fracture, Findakly and Jasim [18] and Kasem et al. [22] reported that MZ crowns with 1 mm shoulder finish lines had more Code V fractures. Similarly, Abdulazeez and Majeed reported that 60% of the MZ crowns with vertical preparations had Code V fractures, and the remaining 40% had either Code III or Code IV fractures [16]. None of them reported Code 1 or Code II fractures. These findings are concurrent with the modes of fractures observed in the present study. Although the data for the fracture resistance of MZ crowns from these studies contradicted our findings, the fracture modes were comparable. This further strengthens our argument that the differences in the fracture resistance observed in this study could be due to the use of ETT as substrate as opposed to non-ETT.

We encountered a few challenges when we classified the fracture modes according to Burke's codes (1999) [25]. The first was the inability to appropriately classify the fracture of the tooth without the fracture of the crown (Grade 3 fractures). We observed such en-mass crown fractures at or below the CEJ in Group 3 samples (Figure 3D). Using Burke's codes, this type of fracture was classified as Code V fracture and could not be differentiated from other types of fractures of the crown or the tooth.

Burke's codes also tended to group restorable and non-restorable fractures of the crown or the tooth into a single entity. Any restorable or non-restorable fracture of the crown or the tooth was just classified as Code V. Thus, we proposed a new grading system, which enabled us to differentiate between the fractures of the crown alone, fractures of tooth and crown, and fractures of the tooth without fracture of the crown. The new grading system also differentiates between restorable and non-restorable fractures. However, this new grading system needs further validation and testing.

Although the shoulder preparations demonstrated lower fracture resistance than BOPT or chamfer finish lines, the observed fracture loads in all the groups were much higher than the maximum masticatory load in the posterior teeth (900 N) [27,28]. This shows that all three finish lines could be used with MZ crowns to restore ETT with clinical success. This is further strengthened by the findings of a clinical trial by El-Ashkar et al., who reported no difference in clinical success between shoulder or feather edge finish lines in ETT with MZ crowns with a follow-up period of one year [29].

This research is subject to certain limitations. One notable limitation is the lack of a chewing simulator prior to the fracture testing. Utilizing a chewing simulator to replicate masticatory loads could cause fatigue failure, which may subsequently affect the fracture loads of the samples. The study was designed as an in vitro investigation, thus making it impossible to achieve an exact replication of clinical conditions. To draw relevant clinical implications, more randomized clinical trials with long-term follow-up are warranted.

## Conclusions

Within the limitations of the present study, it can be concluded that ETT prepared with BOPT and chamfer finish lines had the greatest fracture resistance compared to the shoulder finish line. Teeth prepared with shoulder finish lines had more non-restorable fractures compared to BOPT or chamfer preparations. The shoulder finish line should be avoided on ETT restored with MZ crowns. Conservative tooth preparations such as the BOPT or chamfer finish lines are recommended.
